# A young woman with acute coronary syndrome and antiphospholipid syndrome. Is it the antiphospholipid syndrome or COVID-19 vaccination or classical risk as the risk factor? a case report

**DOI:** 10.1186/s13256-023-04314-0

**Published:** 2024-01-19

**Authors:** Sisca Natalia Siagian, Christianto Christianto

**Affiliations:** 1 Pediatric Cardiology and Congenital Heart Disease Division, Department of Cardiology and Vascular Medicine, National Cardiovascular Center Harapan Kita, Universitas Indonesia, Jakarta, Indonesia; 2 Faculty of Medicine Universitas Indonesia, Jakarta, Indonesia

**Keywords:** Acute coronary syndrome, Antiphospholipid syndrome, COVID-19 vaccination, Risk factor, Young women

## Abstract

**Background:**

Acute coronary syndrome (ACS) in young women is poorly understood due to underdiagnosis and undertreatment. One of the possible risk factors for ACS in young women is antiphospholipid syndrome (APS). Coronavirus disease 2019 (COVID-19) vaccination also emerged as one of the possible risk factors for ACS during the COVID-19 pandemic.

**Case presentation:**

Our patient, a 39-year-old Batak woman with dyslipidemia and family history of cardiovascular disease, experienced chest pain slightly improved at rest accompanied by autonomic symptoms. She was diagnosed with non-ST-elevation myocardial infarction (NSTEMI) based on her clinical features, dynamic electrocardiogram changes, troponin elevation, and multislice computed tomography angiography confirmed with diagnostic catheterization. The patient was managed by percutaneous coronary intervention with implantation of drug-eluting stents. On follow-up, the patient was diagnosed with APS based on history of preeclampsia with severe features in the first and third pregnancy, spontaneous abortion in the second pregnancy, history of transient ischemic attack, moderately positive lupus anticoagulant on two occasions with an interval of 12 weeks, and ACS. Further investigation revealed a history of COVID-19 vaccination with Sinovac four and six weeks before presentation. The patient was recommended for lifelong warfarin and short-term dual antiplatelet (aspirin and ticagrelor).

**Conclusions:**

Young women are not completely immune to ACS as evident in this case of ACS in a young woman with classical risk factors (dyslipidemia and family history of cardiovascular disease) and APS. Further studies are required to fill the knowledge gap on whether COVID-19 vaccination had any contribution to the ACS in the young woman.

**Supplementary Information:**

The online version contains supplementary material available at 10.1186/s13256-023-04314-0.

## Background

Acute coronary syndrome (ACS) is historically regarded as a disease more common in men thus causing its underdiagnosis and undertreatment in women [1]. ACS mainly occurs in elderly patients more than 45 or 50 years old. However, the incidence and prevalence are increasing in the young adult population. Young women with ACS are an especially interesting population given the protective effect of estrogen. The cohort study of young women is uncommon and the risk factors for ACS in young women are poorly understood. [2]

Antiphospholipid syndrome (APS) is an autoimmune systemic disease that is more common in women during their reproductive years compared with men [3]. This condition is characterized by recurrent vascular thromboembolism and/or recurrent fetal loss associated with the occurrence of autoantibodies [4]. Though rarely occurs, ACS is one of the cardiac symptoms that may be found in APS patients [5]. During the coronavirus disease 2019 (COVID-19) pandemic, SARS-CoV-2 infections and vaccinations emerge as the possible causes of thromboembolic complications including ACS. There is an increase in cardiovascular events in young adults after COVID-19 vaccinations [6]. A single-center study also showed that 42% of ACS patients had recent COVID-19 vaccination [7]. We report a rare case of ACS in a young woman with APS and a history of COVID-19 vaccination.

## Case presentation

### Patient information and physical examination

A 39-year-old Batak woman presented to the emergency room of the National Cardiovascular Center Harapan Kita with chest pain. The chest pain was described as a hard suffocating pressure in the middle of the chest lasting more than 30 minute, accompanied by headache, dizziness, vomiting, and profuse sweating. This episode emerged after the patient ran more than 3 km, but the chest pain slightly improved at rest. In the past month, the patient had experienced similar chest pains triggered by strenuous activities. However, the chest pain had been getting worse for the last week as it was triggered even on light activity requiring the patient to take more frequent breaks while working. The risk factors for coronary disease in the patient are dyslipidemia, history of preeclampsia, and family history of cardiovascular disease (details in Table 1).

**Table 1:** Timeline of the case presentation

Time		Events
Family History		• Parents: sudden death at the age of 63 years old caused by CAD and history of myocardial infarction at the age of 40 and 50 years old (father), heart failure and hypertension (mother) • Sudden death at the age of 56 caused by CAD and a history of cardiac arrest at the age of 46 (uncle) • Sudden death at the age of 60 with a history of hypertension and heart failure (aunt)
History of pregnancy complications		• 1st and 3rd pregnancy: preeclampsia with severe features, terminated at 34–35 weeks of pregnancy • 2nd pregnancy: spontaneous abortion at 6–8 weeks of pregnancy
Four years before presentation		• Transient Ischemic Attack (TIA) with symptoms of headache, gait and balance disorder • Clopidogrel 1 × 75 mg for 2 years
Six weeks before presentation		• 1st COVID-19 Vaccination (Sinovac)
One month before presentation		• Chest pain triggered during strenuous activity • 2nd COVID-19 Vaccination (Sinovac)
One week before presentation		• Chest pain triggered during light activity
Initial Presentation	Day 1 (pre-procedure)	• Onset of chest pain after running more than 3 km, slightly improved at rest • Initial assessment revealed NSTEMI • hs-TnT 118 ng/L • MSCT angiography showed significant coronary lesions • Diagnostic catheterization showed diffuse stenosis in proximal-mid LAD with subtotal occlusion in proximal, TIMI 2 flow, and diffuse stenosis in proximal-mid, subtotal occlusion in mid-RCA, TIMI 2 flow • PCI performed with implantation of 2 DES in LAD and 1 DES in RCA
Follow-up	Day 1 (postprocedure)	• Patient was transferred to CVCU with hypotension, given fluid loading and norepinephrine drip down-titrated until stopped completely • After hemodynamic stabilization, the patient was transferred to the ward, and medications were initiated (bisoprolol 1 × 2.5 mg, ticagrelor 2 × 90 mg, aspirin 1 × 100 mg, rosuvastatin 1 × 40 mg, enoxaparin sodium IV 2 × 0.6 cc, lansoprazole 1 × 30 mg)
	4 days	• Enoxaparin sodium discontinued due to bleeding
	5 days	• Patient was discharged
	2 weeks 14 weeks	• Referred to immunoallergy and hematology department with moderately positive lupus anticoagulant • Diagnosed with APS with moderately positive lupus anticoagulant in the immunoallergy and hematology department • Lifelong anticoagulant therapy (warfarin) was recommended

Physical examination revealed a woman with body weight of 62 kg, height of 159 cm, and body mass index of 24.5 kg/m^2^. The vital signs showed high blood pressure of 147/91 mmHg, heart rate of 75 b.p.m., respiratory rate of 20 ×/minute, and oxygen saturation of 100%. Cardiopulmonary and other examinations were within normal limits.

### Diagnostic assessment

**Fig. 1:**
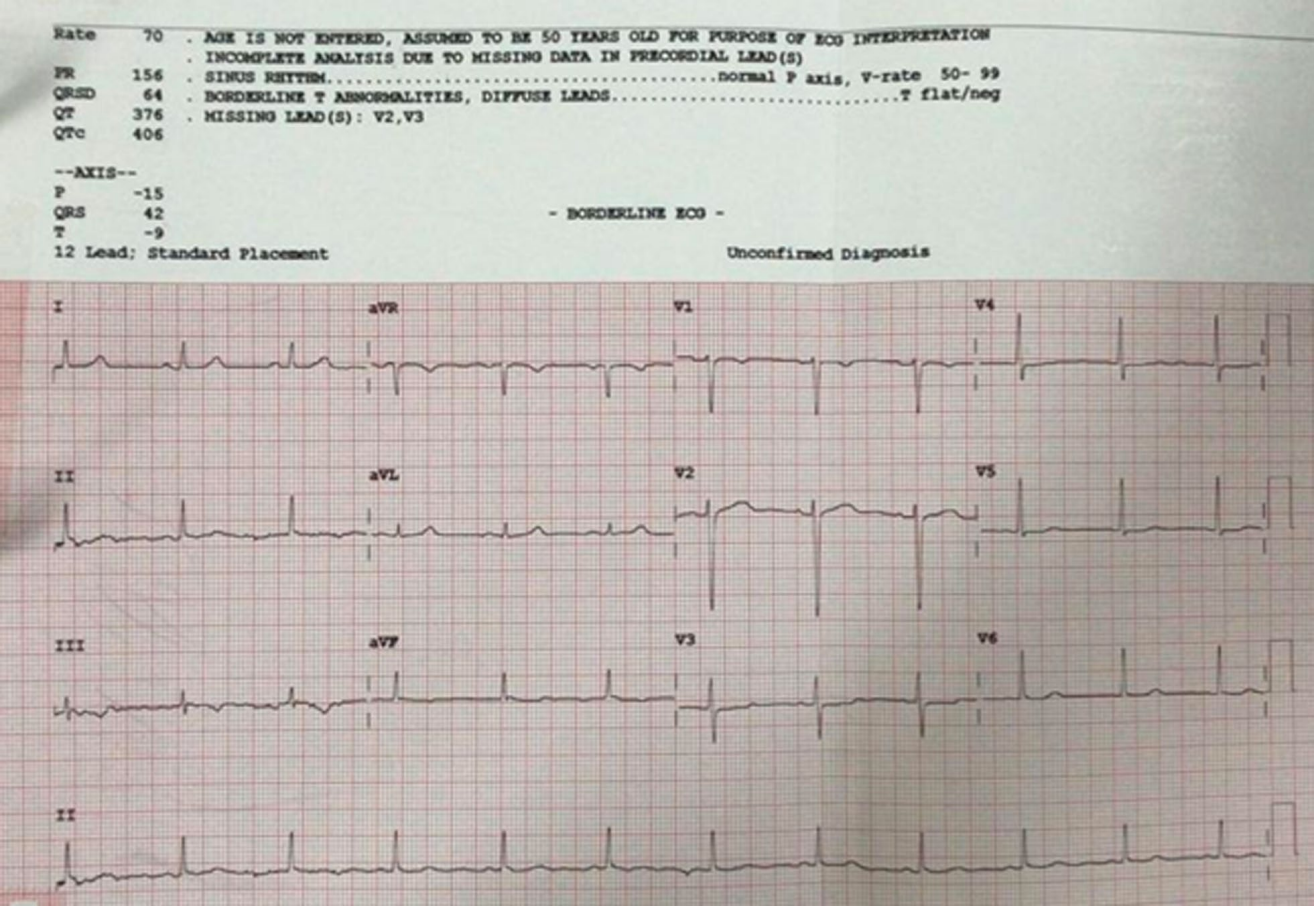
Non-ST-elevation myocardial infarction patterns in the 12-lead electrocardiogram

**Fig. 2:**
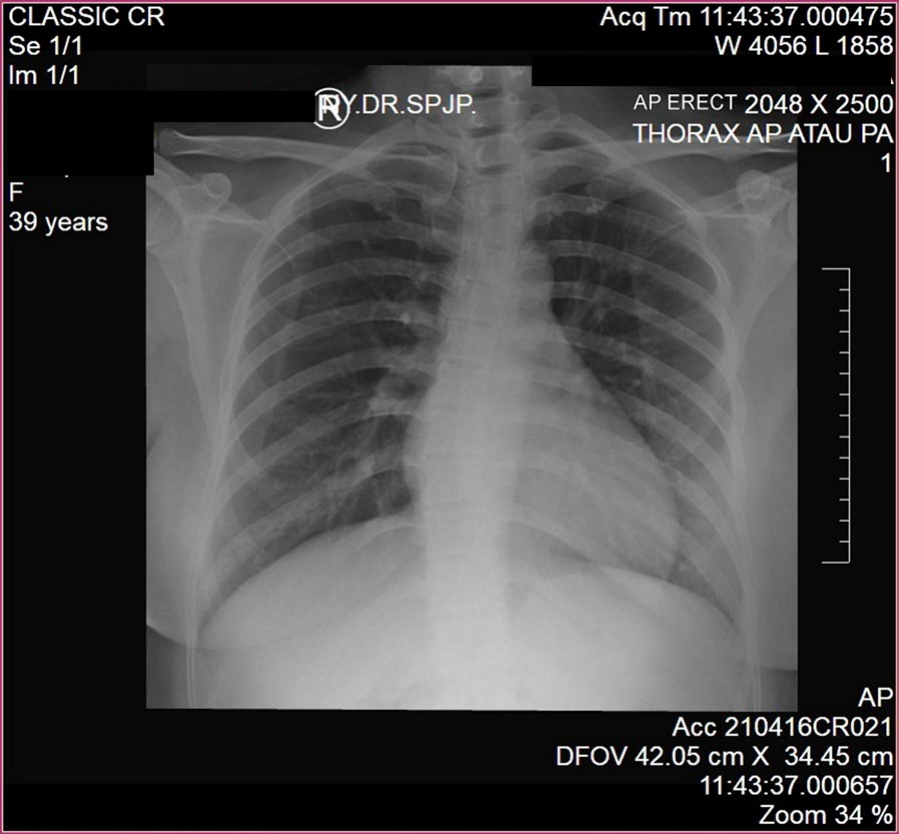
Chest X-ray showing normal radiological finding

Electrocardiogram (ECG) examination showed inverted T wave and slight ST depression in leads III and aVF **(**Fig. 1**)**. Laboratory studies showed increased high sensitivity Troponin T (hs-TnT) of 118 ng/L and Leukocyte of 13,050/μL. Other laboratory studies were within normal limits: blood sugar of 128 mg/dL, eGFR of 106 mL/minute/1.73 m^2^, total cholesterol of 132 mg/dL, LDL of 70 mg/dL, HDL of 50 mg/dL, Triglyceride of 67 mg/dL. The SARS-CoV-2 PCR nasopharyngeal and oropharyngeal swab test was negative. The chest X-ray showed normal radiological findings of the heart and the lungs **(**Fig. 2). Echocardiography findings were within normal limits. The patient also underwent an MSCT angiography examination which showed significant coronary lesions and was advised to be hospitalized. The patient then was diagnosed with non-ST-segment elevation myocardial infarction (NSTEMI) 1/7 GRACE 45 CRUSADE 17 (refractory chest pain).

### Intervention

**Fig. 3:**
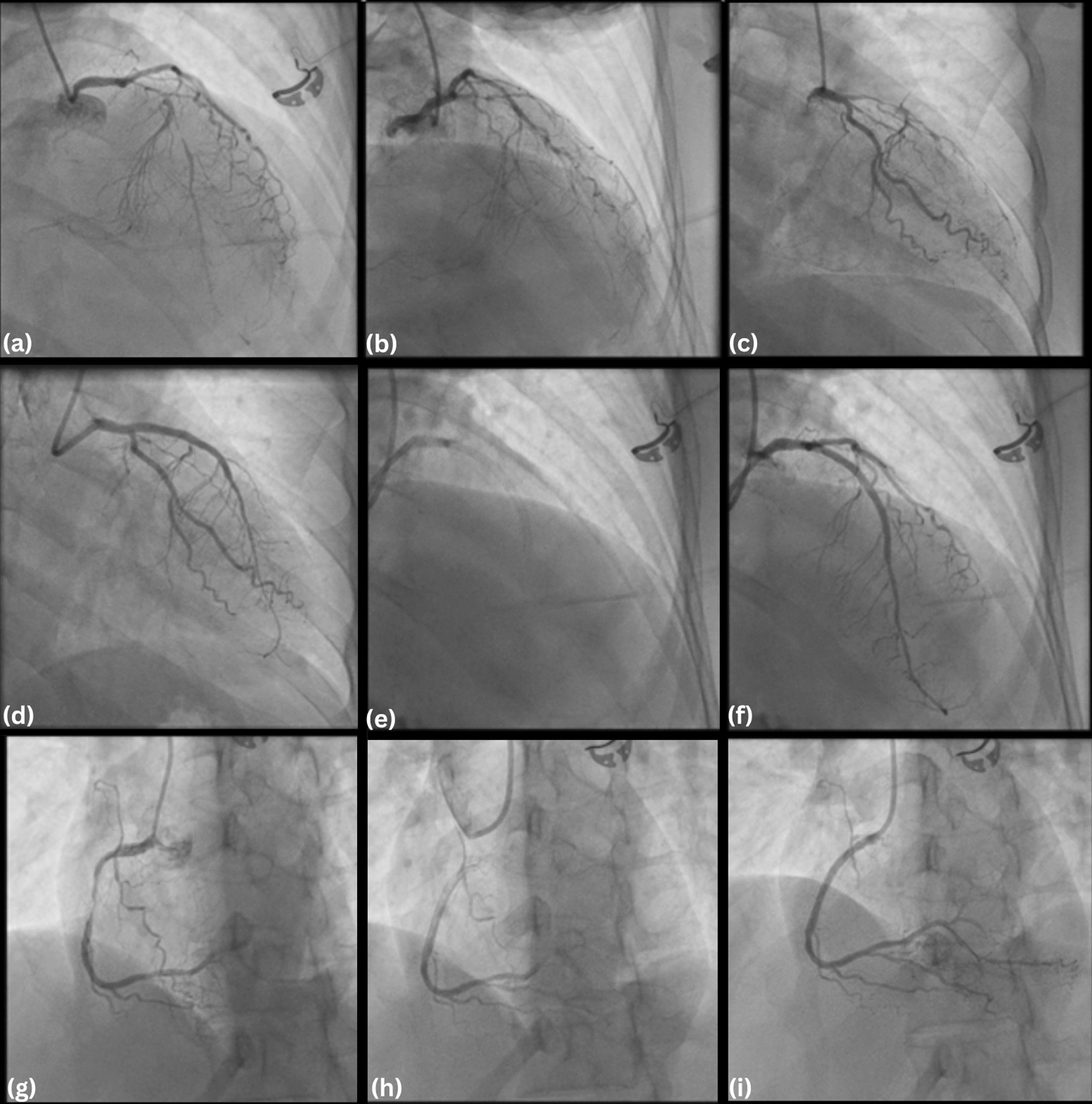
Fluoroscopic percutaneous coronary interventions: **a**–**d** Subtotal occlusion of LAD; **e**, **f** LAD after stenting; **g** Subtotal occlusion of RCA; **h**, **i** RCA after stenting

After consultation with the care team, diagnostic catheterization was conducted showing diffuse stenosis in proximal-mid left anterior descending (LAD) with subtotal occlusion in proximal, TIMI 2 flow, and diffuse stenosis in proximal-mid, subtotal occlusion in mid right coronary artery (RCA), TIMI 2 flow. Percutaneous coronary intervention (PCI) was performed with the implantation of 2 drug-eluting stents (DES) in LAD and 1 DES in RCA (Fig. 3). An additional movie file shows this procedure in more detail (see Additional file 1).

### Follow-up and outcome

After the procedure, the patient was sent to the cardiovascular care unit (CVCU) with hypotension thus fluid loading and continuous infusion of norepinephrine were given. When the blood pressure stabilized, the norepinephrine drip was down-titrated until completely stopped. Following the stabilization of vital signs, further medications were initiated: bisoprolol 1 × 2.5 mg, ticagrelor 2 × 90 mg, aspirin 1 × 100 mg, rosuvastatin 1 × 40 mg, enoxaparin sodium IV 2 × 0.6 cc, and lansoprazole 1 × 30 mg. Enoxaparin sodium was discontinued on day 4 due to bleeding.

The patient was transferred to the ward and discharged on day 5. In addition to the stenosis in the coronary arteries, there was slow coronary flow during the angiography procedure. History taking also revealed a history of preeclampsia with severe features in the first and third pregnancy, both pregnancies terminated at 34–35 weeks of pregnancy. The patient had a spontaneous abortion within 6–8 weeks of her second pregnancy. Additionally, four years before presentation, the patient had a transient ischemic attack (TIA) with symptoms of headache as well as gait and balance disorder. She was then treated with clopidogrel 1 × 75 mg for two years. These findings raised the suspicion of autoimmune disease thus the patient was referred to the immunoallergy and hematology department. The antinuclear antibody (ANA) profile and antiphospholipid antibody (aPL) were negative, except for moderately positive lupus anticoagulant. The patient was tested again 12 weeks after the initial test with a similar result of moderately positive lupus anticoagulant and a ratio of 1.66 which confirmed the antiphospholipid syndrome (APS). Lifelong anticoagulant therapy with warfarin in addition to short term dual antiplatelet (aspirin and ticagrelor) was recommended for the patient. During the evaluation, the history of COVID-19 vaccination with Sinovac four weeks and six weeks before presentation raised the possibility of an adverse event following immunization (AEFI) as it had been reported though in rare occurrence. The timeline of the case presentation is detailed in Table 1.

## Discussion

Although ACS mainly occurs in the elderly, young adults, defined as people with the age between 18 and 45 years old or 50 years in a few studies, can be affected as well [2, 8]. The incidence and prevalence of ACS in young adults, especially young women have been increasing in the past decades to the point that it has been recognized as one of the biggest killers of women. [1, 9] In this case, the patient is a 39-year-old woman which can be categorized into young women population. There were few studies and a lack of awareness of ACS in this population [10]. However, the young women population are unique as studies showed different pathophysiologic mechanisms, different risk factors, worse prognosis, higher morbidity, and mortality of ACS compared with man and older populations. [1, 9, 10]

Studies reported the risk factors of ACS in young women such as smoking, hypertension, dyslipidemia, abdominal obesity, diabetes, hypercoagulable state (hormonal therapy, antiphospholipid syndrome, nephrotic syndrome, factor V Leiden gene mutation), menopause, and family history of one parent and stroke (hemorrhagic and ischemic) [1, 8, 11, 12]. A meta-analysis reported diabetes mellitus, hypertension, and hypercholesterolemia as the strong independent risk factors of ACS in young women [13]. The patient had several known risk factors of ACS which were dyslipidemia, family history of cardiovascular disease, and recently diagnosed APS (history of severe preeclampsia and TIA). In the COVID-19 pandemic, COVID-19 vaccination and infection emerged as the risk factors of ACS in young adults because of increased thromboembolism complications [6, 7]. However, COVID-19 infection can be ruled out based on the clinical, laboratory, and radiological examinations leaving COVID-19 vaccination as one of the possible risk factors of ACS in the patient.

Dyslipidemia is an established risk factor for ACS related to atherosclerosis in men and the elderly group. [14, 15]. This is also the case in young women with ACS who have a high incidence of dyslipidemia [16]. Dyslipidemia was an independent predictor of ACS in the young women group with a relative risk (RR) of 23.94 (95% CI 12.209–46.943), while specifically hypercholesterolemia increased the risk of ACS by 3.45–5.23 [8, 17]. In this case, the patient’s total cholesterol, triglyceride, LDL levels, and HDL were within normal limit as the patient had been taking atorvastatin 20 mg once daily to control her dyslipidemia. Therefore, dyslipidemia was less likely to be a dominant risk factor of ACS in the patient even though it might still contribute to the progression to ACS in one way or another.

Certainly, family history plays a significant role in the case of ACS in young women [11]. The sudden death from CAD with a history of myocardial infarction of the patient’s father was the most significant family history (first-degree relative) related to the increased risk of ACS in the patient. RATIO study reported that women with at least one parent who suffered from myocardial infarction are at four times higher risk of ACS [18]. The uncle’s sudden death from CAD with a history of cardiac arrest and the aunt’s sudden death with a history of hypertension and heart failure, as well as the mother with heart failure and hypertension cannot be excluded as family history which may contribute to the risk factor of ACS in the patient. One study reported an association of family history of CAD with ACS in young women (OR 1.62, 95% CI 1.35–1.94) [19]. Therefore, the family history of cardiovascular disease was one of the risk factors for ACS in the patient.

Nonetheless, this case was unique as the patient had a history of preeclampsia with severe features in the first and third pregnancy, both pregnancies terminated at 34–35 weeks of pregnancy, and first-trimester miscarriage in the second pregnancy. The finding of moderately positive lupus anticoagulant and the pregnancy history of the patient in addition to the ACS confirmed the diagnosis of APS. The occurrence of ACS in APS has been known since the syndrome was first described and may even be the first presentation of APS [4]. APS is usually accompanied by a hypercoagulable state caused by the antiphospholipid antibody (aPL) which induces upregulation of adhesion molecules in endothelial cells, increased platelet adhesion, and inhibition of anticoagulant and fibrinolytic pathway. The association between aPL and ACS is more frequent in women. [20]

While APS was indeed a risk factor for ACS in the patient, the magnitude of APS-induced thrombosis in the patient was unclear. In the RATIO (Risk of Arterial Thrombosis In relation to Oral contraceptives) study, the odds ratio for ACS in patients with lupus anticoagulant was 5.3 (95% CI 1.4–20.8) [21]. However, there were discrepancies regarding the association between each aPL subtype and ACS. Therefore, the association of aPL and the increased risk of ACS is still a matter of controversy. This may be due to the classification of APS which does not exclude the coexisting risk factors for thrombosis. [4]

The adjusted Global APS Score (aGAPSS), a scoring model based on five clinical factors (three aPL specificities, arterial hypertension, and hyperlipidemia), is a tool to assess the thrombosis or pregnancy loss risk in APS [22]. Studies showed that the aGAPSS was also useful for risk stratification of ACS occurrence in young APS patients with thrombotic events as well as the risk of developing recurrent thrombosis in APS [22, 23]. A few studies used the cut-off value of 10 for the high-risk group. A higher value of aGAPSS correlates with a higher risk of first or recurrent thrombosis event [24, 25]. In this case, the patient had hyperlipidemia and positive lupus anticoagulant resulting in a total aGAPSS of 7 thus the patient was not in the high-risk group. There is a concept that aPL is a necessary but insufficient step in the development of thrombosis. A second hit, consisting of other risk factors, probably pushed the hemostatic balance in favor of thrombosis [22, 26]. Nonetheless, even if the patient was not in the high-risk group, APS is still a contributing factor in the occurrence of ACS in the patient.

During the COVID-19 pandemic era, there have been reports of myocardial infarction postvaccination with the Pfizer, AstraZeneca, and Sinovac vaccines. The gap between vaccination and the occurrence of ACS varied from 15 minute to 2 days. There are several mechanisms of myocardial infarction induced by vaccination: immune thrombotic thrombocytopenia, a series of allergic reactions which lead to occlusion of coronary arteries (Kounis syndrome), and high demand and low supply due to vaccination stress in weaker patients [27]. Vaccine-induced immune thrombotic thrombocytopenia (VITT) was more commonly related to adenoviral vector vaccine typically 5–30 days postvaccination. Although there were reports of thromboembolic events in various COVID-19 vaccine types, most of them were associated with mRNA [27].

There was no adverse cardiac event in the initial report from the randomized, phase I/II, double-blind, placebo-controlled CoronaVac vaccine trial with healthy adult subjects aged 18–59 years [28]. However, there were rare cases of ACS postvaccination with inactivated vaccines such as CoronaVac, even though the causal relationship cannot be established. Some of the hypotheses of the mechanisms of inactivated vaccine-induced ACS were psychological factors which increased myocardial oxygen demand (type 2 MI), coronary vasospasm, inflammation process related to the immune response to vaccination which may aggravate the coronary plaque to rupture, and increased thrombotic event [29]. One study reported that COVID-19 vaccinations were well tolerated in APS patients which showed that there might be no interaction between both factors [30]. The laboratory studies of the patient showed normal thrombocyte level thus excluding VITT in the patient. The patient was vaccinated with Sinovac which is an inactivated COVID-19 vaccine with a period of four weeks before presentation. While it was less likely that the COVID-19 vaccination had a dominant impact on the occurrence of ACS in the patient, there might still be a possibility of it being a contributing factor of ACS in the patient. In this case, the initial pathology was most likely atherosclerosis of the coronary arteries as the patient had symptoms one month before the occurrence of the ACS. It was also supported by the fact that the patients had many risk factors for the progression of coronary artery disease. We believed that the pathophysiology of ACS in this patient was a thrombotic event as supported by the angiography showing diffuse stenosis of the coronary arteries in addition to the fact that there was no supporting risk factor for an embolic event such as atrial fibrillation. APS indeed increased thrombogenicity causing ACS in the patient, but the contribution of COVID-19 vaccination in the occurrence of ACS in this case was less likely.

Regardless of which risk factors were more dominant, the patient had APS which is a clinical challenge in the management of the ACS. The patient had been managed with PCI with implantation of DES. APS patient who undergoes stent implantation in the culprit lesion is recommended to be given triple antithrombotic therapy with short-term dual antiplatelet therapy and long-term anticoagulation while balancing the risk of stent thrombosis and increased risk of bleeding. Vitamin K antagonist (VKA) as anticoagulation should be administered for life as secondary thrombosis prevention due to the extremely high risk of recurrent thrombotic events [31]. Warfarin at an INR > 3.0 or low-dose aspirin in addition to standard-intensity warfarin (2.0–3.0) is the current recommendation. However, some experts believe that antiplatelet therapy alone or warfarin with an INR range of 2.0–3.0 is sufficient. In recurrent thrombosis, a higher target INR should be achieved [4]. The use of novel oral anticoagulants (NOACs) is not yet established in patients with APS. Direct oral anticoagulants are not recommended as they are less effective and less safe than vitamin K antagonists for long-term thromboembolism prevention in APS patients [31, 32]. Therefore, the patient was recommended for long-term anticoagulant therapy with warfarin in addition to short-term dual antiplatelet therapy (aspirin and ticagrelor).

## Conclusions

Young women are not completely immune to ACS as evident in this case of ACS in a young woman with classical risk factors (dyslipidemia and family history of cardiovascular disease) and APS as the risk factors of ACS. Further studies are required to fill the knowledge gap on whether COVID-19 vaccination had any contribution to the ACS in the young woman.

### Supplementary Information

**Additional file 1. **Percutaneous coronary intervention in a young woman with ACS and antiphospholipid syndrome.

## Data Availability

Not applicable.
